# Toxic plants act as indiscriminate protectors of insects

**DOI:** 10.1038/s42003-021-02830-7

**Published:** 2021-11-16

**Authors:** Luke R. Grinham

**Affiliations:** Communications Biology, https://www.nature.com/commsbio/

## Abstract

Aposematism is a prey strategy to communicate toxicity or danger to predators, often through bright colours, and over time is learned by predators. McLellan et al. report in *Current Biology* that association between an aposematic insect and its host plant is learned by wild birds, to the point that any insect on the plant faces a lower predation risk.

## Main

Aposematic signalling is prevalent across many animal and plant species, and is a prey strategy to survive predation pressure. Bright colours, sounds or odours signal to predators that eating the target is not going to be an easy, pleasant or nutritious meal.

**Fig. 1:**
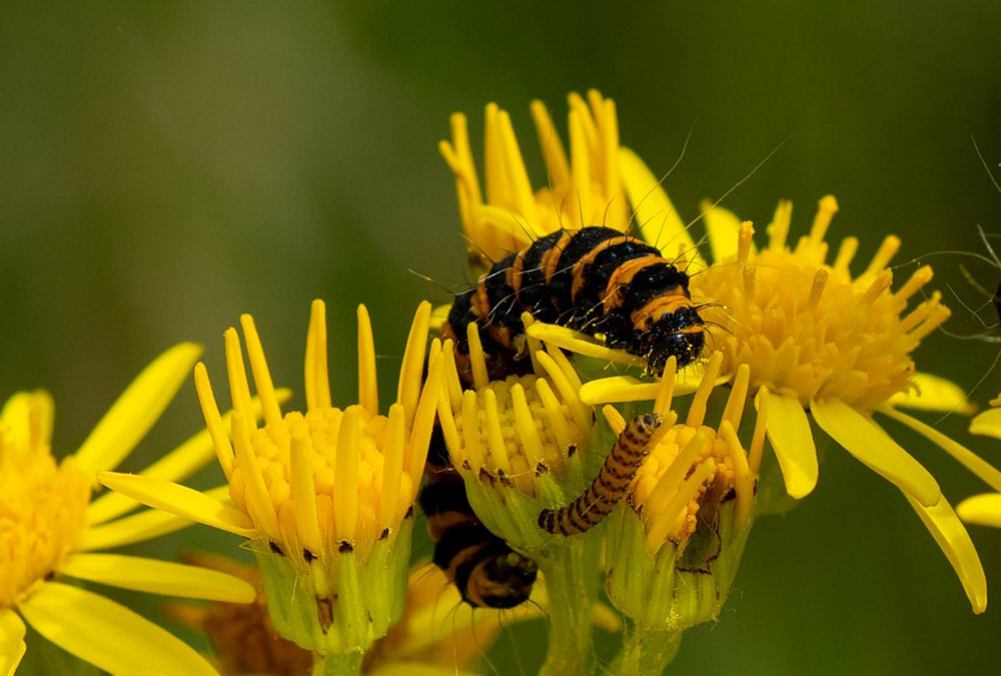
Cinnabar moth larva on ragwort flower. Image by Mark Underwood from Pixabay.

For cinnabar moth larvae, aposematic signalling via black and orange stripes (Fig. 1) facilitates safe growth to adulthood. The caterpillar feeds exclusively on ragwort, a plant with bright yellow flowers that is toxic to most vertebrates. Callum McLellan et al.^1^ from the University of Bristol, UK, showed that dummy caterpillars (‘targets’) placed on ragwort plants are at a lesser predation risk from birds than those on other plants, even when the targets themselve lack the orange and black warning stripes of the cinnabar moth larvae. They also found that naive predators, such as juveniles, do not make this association. Instead, these inexperienced predators hunt all targets on all plants at about the same rate. This shows that the recognition of both aposematic prey and the plant association are learned behaviours in a natural environment.

Based on these findings, the authors suggest that the aposematic phenotype of the cinnabar moth larvae has been extended to the host plant as a type of guilt by association. Predators learn that the toxic, brightly coloured insects are found on a specific host plant and may therefore avoid any insects on that plant, which may also be unpalatable. Theoretically, such associations could be exploited by other prey species in the same ecosystem, broadly benefitting their survival under predation, thanks to a single species’ close association with a host. At the same time, this could reduce the selective pressure of aposematic signalling in the prey species, as the aposematic message is no longer dependent on its own signal but rather that of its host. Given the costs that are often associated with bright colouration, such as being easily spotted (which clearly serves no defence against naive predators), this could in turn confer a future evolutionary benefit. Exploring this association in more systems should be a future target for the researchers studying predator–prey interactions, particularly in mammalian and reptilian systems, or in instances where harmless prey take refuge alongside more dangerous hosts.
